# Cellular origins and lineage relationships of the intestinal epithelium

**DOI:** 10.1152/ajpgi.00188.2021

**Published:** 2021-08-25

**Authors:** Claudia Capdevila, Maria Trifas, Jonathan Miller, Troy Anderson, Peter A. Sims, Kelley S. Yan

**Affiliations:** ^1^Columbia Stem Cell Initiative, Division of Digestive and Liver Diseases, Department of Medicine, Columbia Center for Human Development, Columbia University Irving Medical Center, New York, New York; ^2^Department of Genetics & Development, Columbia University Irving Medical Center, New York, New York; ^3^Department of Systems Biology, Columbia University Irving Medical Center, New York, New York; ^4^Department of Biochemistry & Molecular Biophysics, Columbia University Irving Medical Center, New York, New York

**Keywords:** differentiation, intestinal epithelium, intestinal stem cells, lineage hierarchy, single-cell RNA-sequencing

## Abstract

Knowledge of the development and hierarchical organization of tissues is key to understanding how they are perturbed in injury and disease, as well as how they may be therapeutically manipulated to restore homeostasis. The rapidly regenerating intestinal epithelium harbors diverse cell types and their lineage relationships have been studied using numerous approaches, from classical label-retaining and genetic lineage tracing methods to novel transcriptome-based annotations. Here, we describe the developmental trajectories that dictate differentiation and lineage specification in the intestinal epithelium. We focus on the most recent single-cell RNA-sequencing (scRNA-seq)-based strategies for understanding intestinal epithelial cell lineage relationships, underscoring how they have refined our view of the development of this tissue and highlighting their advantages and limitations. We emphasize how these technologies have been applied to understand the dynamics of intestinal epithelial cells in homeostatic and injury-induced regeneration models.

## Graphical abstract

**Figure:**
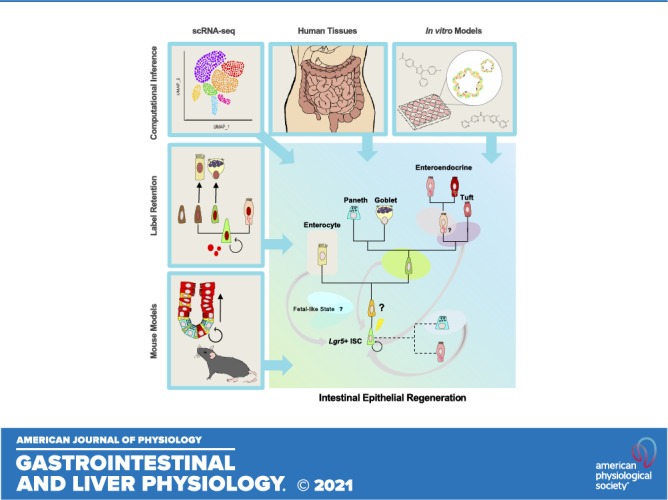

## INTRODUCTION

Understanding cell lineage relationships is a fundamental goal of stem cell and developmental biology. From the very early decisions undertaken by the developing embryo to the ones that govern adult tissue homeostasis, the maintenance of cellular hierarchies and the proper balance between coexisting populations is crucial for normal tissue development and function. Dysregulation of these processes is associated with developmental disorders, aging, and tumorigenesis. Thus, elucidating the hierarchical organization of tissues is key to understanding how these become impaired during injury and disease and how they can be manipulated to reinstate homeostasis. Furthermore, the mechanisms underlying lineage decision can inform directed differentiation in stem cell therapy approaches (1, 2), where cells from one lineage are derived at will from other cell types to aid tissue regeneration, rescue tissue functions that have been lost, or provide compensatory functions to ameliorate an impairment.

Much of our current knowledge of lineage relationships has been gained through lineage tracing approaches that reveal the fates of individual cells by examining the identities of their progeny. Lineage tracing technologies have evolved over the years from early cell labeling-based observational methods to the current single-cell RNA-sequencing (scRNA-seq)-based algorithms for lineage reconstruction (3–5). These approaches have been particularly insightful for understanding rapidly regenerating tissues such as the intestinal epithelium. This tissue comprises highly diverse cells that carry out its diverse effector functions, most notably nutrient absorption, immune barrier function, and the secretion of mucin and a wide variety of hormones that regulate systemic metabolism (6). Despite decades of study, the roadmap showing the developmental history of differentiated cell types remains poorly understood. Many of the developmental intermediates and their fate decisions along the numerous developmental trajectories remain unclear.

## OVERVIEW OF THE INTESTINAL EPITHELIAL LINEAGES: IS IT TIME TO REVISIT THE MODEL?

**Figure 1.**
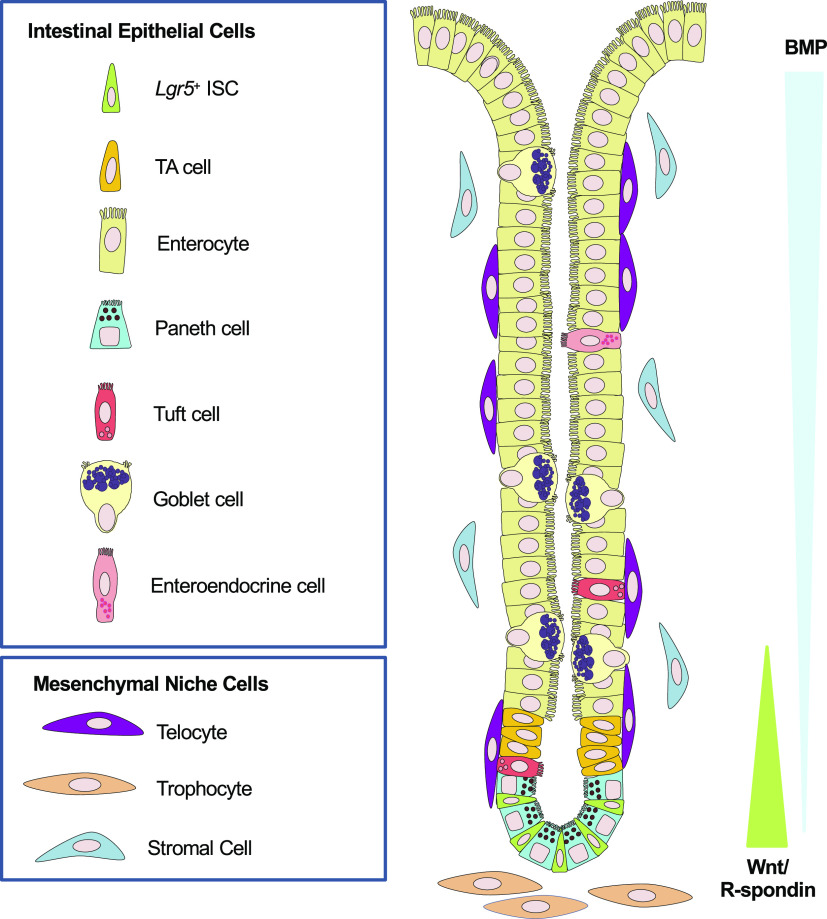
Overview of the intestinal epithelium and its intestinal stem cell (ISC) niche. The small intestine is organized into proliferative crypt compartments and villi. The crypts harbor *Lgr5*^+^ ISCs that give rise to the major intestinal lineages including absorptive enterocytes, mucus-producing goblet cells, chemosensory tuft cells, hormone-producing enteroendocrine cells, and Paneth cells. Within the epithelial lineage, Paneth cells elaborate (nonessential) Wnt, EGF, and Notch signals that influence ISCs. Surrounding the intestinal epithelium, mesenchymal cells secrete niche factors to orchestrate Wnt/R-spondin signaling near the crypt base and BMP gradients to influence differentiation along the crypt-villus axis. BMP, bone morphogenetic protein; ISCs, intestinal stem cells; TA, transit-amplifying cell.

The intestinal epithelium is maintained by a cycling population of crypt-base columnar (CBC) intestinal stem cells (ISCs) that are characterized by expression of the R-spondin (Rspo) receptor *Lgr5* (6–8) and their functional capacity for long-term self-renewal and multilineage differentiation into both absorptive and secretory lineages (6–15). *Lgr5*^+^ ISC self-renewal is dependent on the presence of Wnt and Rspo (16), two factors that are mainly secreted by a heterogeneous population of crypt-adjacent subepithelial mesenchymal cells constituting the stem cell niche (Fig. 1) (17–21). The localization of supportive niche factors in the crypt provides an environment favorable to the maintenance of the stem cell state, and alterations in these self-renewal signals along the crypt-villus axis result in differentiation (6, 8, 9, 19–22). During homeostasis, multiple ISCs support intestinal epithelium turnover via neutral drift kinetics, whereby multiple clones compete for niche space (23, 24). Although recent mathematical simulations contemplate the possibility of asymmetric ISC division (25), the prevailing model states that ISC divisions result primarily in symmetric daughter cells whose fates are either supported by the permissive environment of the crypt to maintain stemness or become displaced from the niche and undergo differentiation as they exit the crypt (23, 24, 26). Therefore, in the intestinal epithelium, the balance of self-renewal to differentiation is remarkably controlled at the population level rather than the single-cell level, with those cells in spatial proximity to the niche boundary having a higher propensity to undergo differentiation (26).

Differentiation in the intestinal epithelium is thought to occur at the “origin of differentiation” or “+5” cell position, where cells choose between absorptive and secretory fates (6, 8, 15). Differentiation is broadly defined by the progressive loss of self-renewal and multipotency coupled to the acquisition of mature cell features, embodied by a series of intermediate or progenitor cell states with increasingly restricted lineage competence. Transit-amplifying (TA) cells are thought to occupy the upper crypt zone that is at the interface between self-renewal and differentiation, and they are historically considered as the first step an ISC needs to take along its path to differentiation (6). According to their operational definition, TA cells proliferate rapidly and are able to renew for several divisions, but eventually undergo multilineage differentiation and hence they become exhausted unless actively replenished by the *Lgr5*^+^ ISC pool (6). During this process of differentiation, maturing cells move rapidly up the crypt-villus axis, ostensibly fueled by the extensive proliferation in the crypt and active sorting via Eph-Ephrin signaling (8). Conversely, Paneth cells localize to the crypt base to interdigitate between the ISCs where they secrete antimicrobial products and nonessential self-renewal factors (27, 28). In the colon, Paneth cells are absent and potentially substituted by a rare *Reg4*^+^ deep crypt secretory cell with analogous niche-associated roles (29, 30). In some scRNA-seq studies, TA cells are identified a priori by their proliferative gene expression program and lack of an *Lgr5*^+^ stem-cell-specific gene signature (16, 31, 32). Interestingly, intestinal TA cells have continued to elude a molecular definition because there currently is a lack of specific, validated markers for their prospective isolation. Indeed, the existence of such a multilineage-primed TA population has been called into question (8, 33–36). Considering this, their transient nature, and the fact that important fate decisions are likely made immediately downstream of the ISC, one can understand why little is known about the developmental trajectories of the intestinal epithelium—starting from the fundamental question as to whether TA cells exist or not.

Studies using biochemical and genetic perturbations have yielded important principles of hierarchical organization. As a consequence, other aspects of intestinal epithelial lineage specification are better defined. Although disruption of the Wnt/Rspo axis unleashes ISCs from the stem cell state (16), Notch signaling is critical to bias cell fate toward the absorptive lineage in a second fate decision (8, 37, 38). Biochemically, this is achieved by blocking secretory cell differentiation through the antagonistic roles of HES1 and ATOH1 transcription factors (37, 39). Upon activation of Notch receptors by either adjacent DLL1/DLL4-expressing Paneth cells or secretory progenitors, ISC/TA cells express HES1, whose two main roles include *1*) repression of CDK inhibitors p27 and p57, hence favoring cell division and *2*) repression of ATOH1, which maintains Notch ligand expression in the plasma membrane and constitutes a master regulator for secretory fate specification. Thus, a progenitor that loses access to Notch ligands will upregulate DLL1/DLL4 on its surface and become secretory biased, subsequently supporting Notch activation in surrounding progenitors to specify the enterocyte fate (8, 9, 37, 38). This phenomenon, known as lateral inhibition, amplifies and stabilizes stochastic differences in Notch pathway activation, translating them into robust fate decisions. In parallel, through its cell cycle control, this system ensures that the ratio of absorptive to secretory progeny is skewed toward enterocyte production, since secretory progenitors are rendered postmitotic.

**Figure 2.**
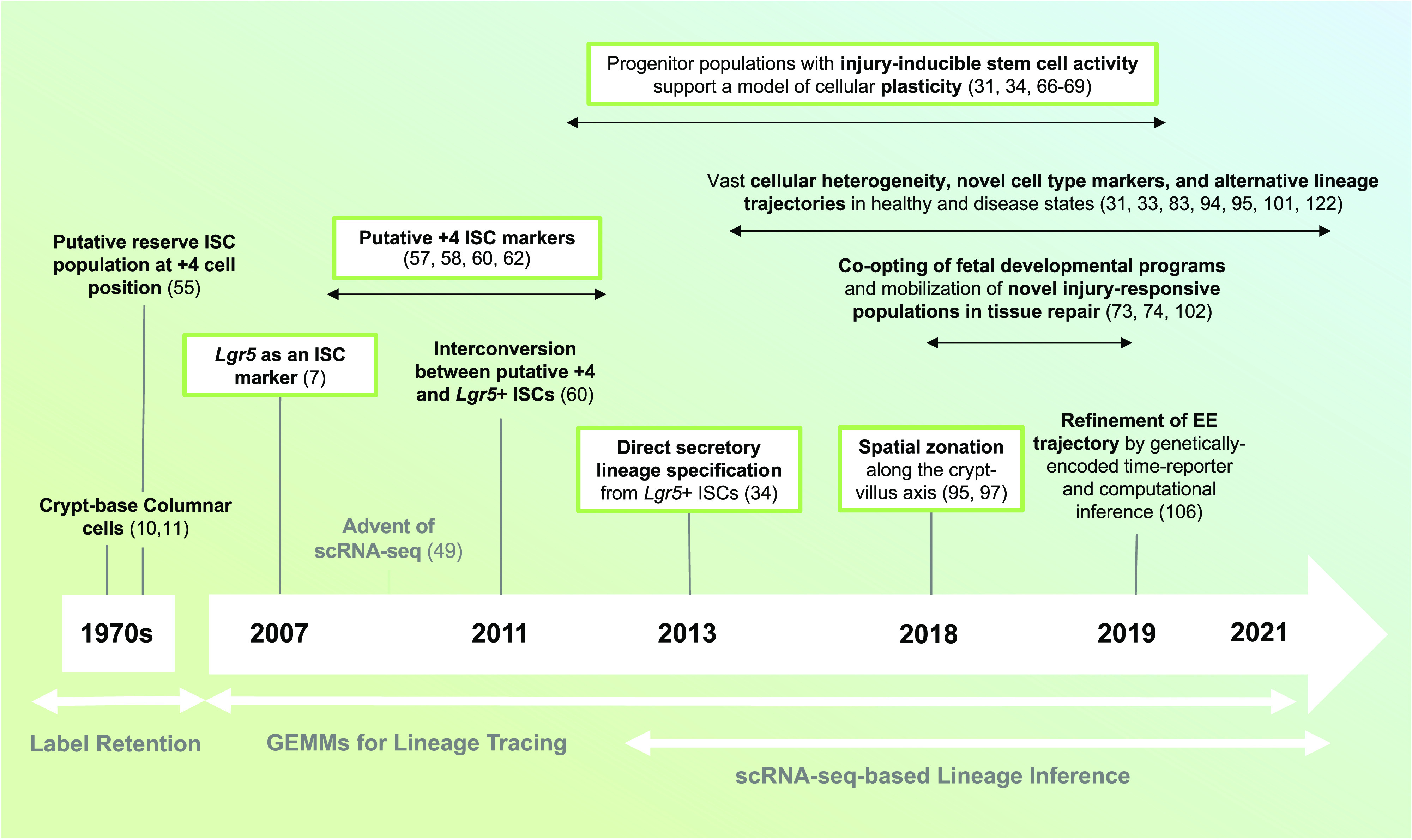
Timeline for how the evolving lineage reconstruction techniques have reshaped our understanding of intestinal epithelial regeneration. Although genetic lineage tracing has been instrumental in the definition of individual populations of stem cells and progenitors, the advent of single-cell RNA-sequencing (scRNA-seq) (49) expanded our understanding on the cellular heterogeneity of this tissue and the dynamic relationship between the stem cell compartment and its lineages. scRNA-seq has also been instrumental for helping to uncover alternative lineage origins and the importance of cellular plasticity in intestinal epithelial repair following injury. GEMMs, genetically engineered mouse models; ISCs, intestinal stem cells.

**Figure 3.**
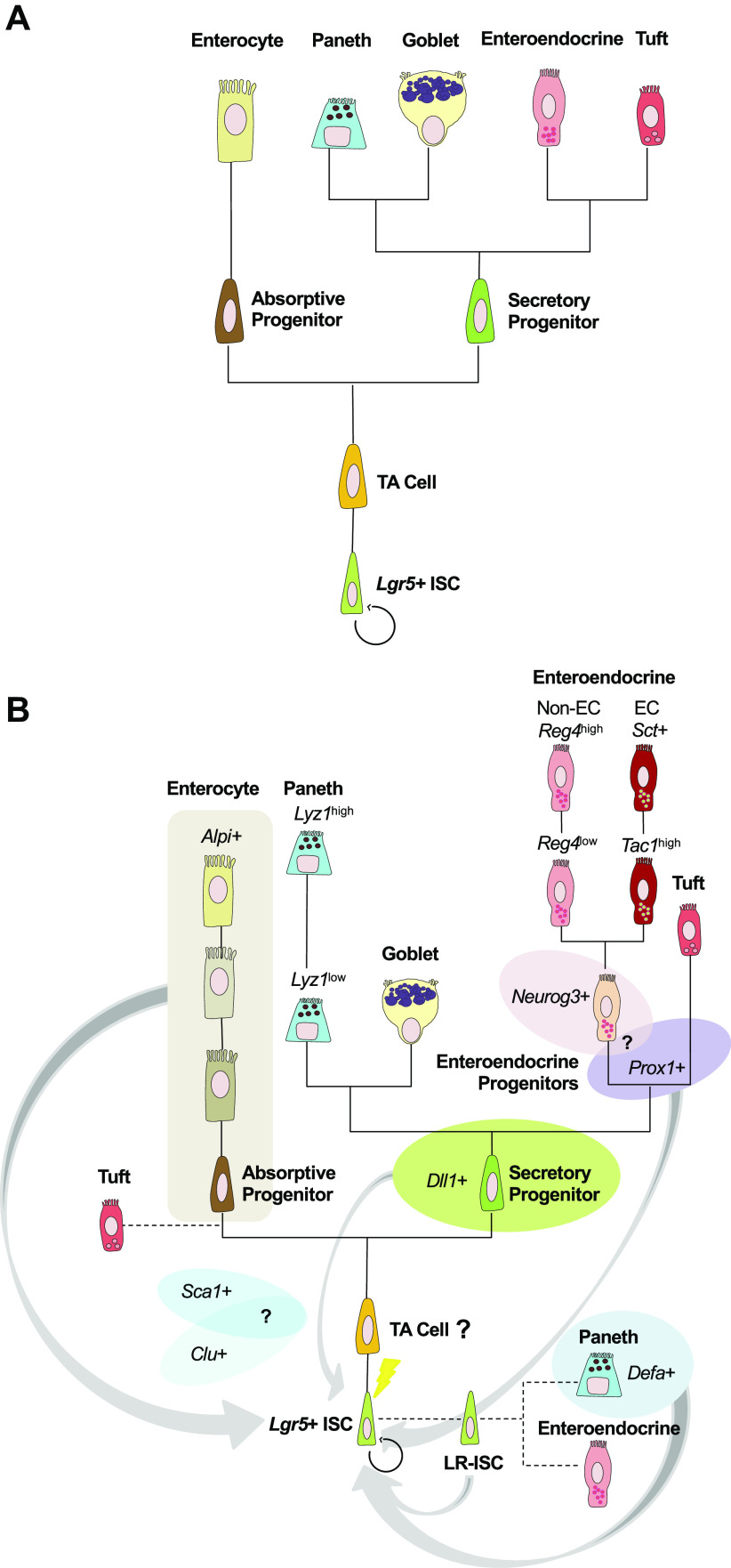
Classical and refined models of the intestinal epithelial lineage hierarchy. *A*: previous understanding of the intestinal lineage separated absorptive and secretory progenitor lineages. *B*: refinement of this model demonstrates plasticity between epithelial cells. Diversification along the enteroendocrine lineage is shown, exemplified by enterochromaffin (EC) cells of different expression profiles and non-EC *Reg4*^high^ and *Reg*^low^ cells. Enterocyte maturation stages are also depicted, as these carry out specific functions as they differentiate. Alternative lineage origins are also shown here, including label-retaining (LR)-ISCs giving rise to Paneth and enteroendocrine cells, and nonconventional tuft cell origins. Finally, note the inclusion of injury-responsive populations (gray arrows), including potentially overlapping, newly defined *Sca1*^+^ and *Clu*^+^ populations at an undefined position within the lineage. ISCs, intestinal stem cells; TA, transit-amplifying cell.

Eventually, the selection of the specific cell type that a progenitor becomes is dependent on the expression of one or several master regulator transcription factors, as dictated by multiple signaling cues [most notably, varying levels of Notch, Wnt, and EGF signals; see Beumer et al. (9) for an excellent review on the topic]. This was demonstrated by distinct null and conditional loss-of-function (LOF) animal models, which facilitated the decoding of the genetic requirements for the specification of the intestinal epithelial cell lineages and suggested the existence of shared intermediate cell types, hence approximating lineage reconstruction in the intestine (8). For example, the essential *Neurog3* requirement for enteroendocrine (EE) cell specification was demonstrated both in a *Neurog3* null mouse model (40) and later confirmed in humans through genome screenings (41). Similarly, one report showed *Gfi1* is required for Paneth and goblet cell generation and highlighted its role in a putative mutually exclusive EE versus goblet/Paneth cell fate decision through a common granulocytic progenitor (42). Subsequent studies refuted the existence of such a precursor, ascribing prior results to the general repressive role *Gfi1* exerts on *Neurog3* (43). This exemplifies some of the experimental challenges of LOF and overexpression approaches for lineage tree inference. Similar studies have attempted to address cellular origins of tuft cells. These DCLK1^+^ chemosensory cells initiate type II immune responses against parasitic infections and were initially considered an EE subtype (44–46). However, extensive marker profiling and LOF assays demonstrated that these do not belong to any of the identified epithelial lineages and should be considered a separate secretory type (46). Together, these and other data suggest a paradoxical Notch-repressed, *Atoh1*-independent mechanism for tuft cell specification, although *Atoh1* indispensability is not embraced by all (46–48). As such, the ontogeny of tuft cells remains controversial. These unanswered questions highlight the need for novel approaches to fill our knowledge gaps. As discussed below, this prototypic intestinal lineage tree has been questioned and shaken to its roots by new and more precise technologies (Figs. 2 and 3).

## LINEAGE RECONSTRUCTION IN THE INTESTINAL EPITHELIUM: A FOCUS ON SINGLE-CELL RNA-Seq-BASED APPROACHES

The properties of long-term self-renewal and multilineage differentiation have served to operationally define adult stem cells and distinguish them from their immediate TA and early progenitor progeny. However, in some tissues, such as in the hematopoietic system, these properties have been assigned on the basis of transplantation and colony-formation assays (50, 51), in which the fate of individual cells is evaluated following removal from their native instructive niches (52). Lineage tracing is a technique that allows for the direct mapping of a founder cell’s progeny, in vivo and ideally at single-cell (clonal) resolution, hence bypassing these limitations (3–5). If carried out during homeostasis, it can reveal how tissue hierarchies are structured under physiologic conditions, and if performed under an injury-repair setting, it can serve to delineate how a system adapts or repurposes its available lineages to cope with different stressors. This approach is a step forward compared with lineage inference based on transplantation and in vitro assays. It constitutes an important deviation from the perturbational approaches discussed above, which indirectly probe the mechanisms of fate determination by measuring compositional changes in cell types after genetic mutation, overexpression, or cell-signaling interference.

Prospective lineage tracing approaches all rely on the passage of a readily identifiable genetic label from a cell to its progeny (4), and the resultant range of cell types observed in the descendants and their long-term persistence informs on the lineage potential and self-renewal capacities of the original labeled cell. In its stricter definition, lineage tracing lacks spatial resolution and therefore cannot inform on positional information of the various tracked lineages (53). Although spatial allocation has traditionally been the job of fate mapping, nowadays the boundaries between lineage tracing and fate mapping intertwine, with current lineage tracking schemes allowing for clonal or subclonal labeling and incorporation of spatial visualization into a cell’s progeny (53). For simplicity and for historical reasons, we will refer to these prospective methods as lineage tracing, even if they do not necessarily fit the clonal labeling requirement of early clonal analysis (53). As technologies evolve, prospective lineage tracing has been complemented by alternative approaches for lineage tree inference. A summary of how some of these evolving techniques have helped reshape our understanding of the intestinal epithelial lineage relationships is shown (Fig. 2).

Prospective lineage tracing techniques have served to delineate the intestinal epithelial lineage and to identify its potential stem cell sources, first through transient radioisotope labeling that led to the description of the crypt-base columnar cell (CBC) (10, 11), subsequently identified as the *Lgr5*^+^ ISC (7), and around the same time through a long-term labeling strategy with nucleotide analogs to report the existence of a putative quiescent, reserve ISC population at the +4 position (54, 55). The idea of a slow-cycling, reserve stem cell population that gets mobilized upon tissue damage is attractive, especially considering that rapidly cycling *Lgr5*^+^ ISCs are dispensable during homeostasis (56) and seemingly at odds with the attributes of relative rarity and quiescence of other well-studied stem cell populations such as the hematopoietic stem cell. Numerous and potentially overlapping label-retaining/quiescent populations have been proposed, identifiable by markers like *Bmi*, *mTert*, *Lrig1*, and *HopX* (56–62). However, several contradictory reports have also been published regarding the identity, heterogeneity, and “professionalism” of the +4 ISC (63). Indeed, some of these genes are promiscuously expressed and can be found in both *Lgr5*^+^ ISCs as well as in committed progenitor and differentiated cell types (31, 44, 64). As a consequence, the degree of overlap and the possibility of interconversion between slowly and rapidly cycling ISCs remain intriguing (60, 65).

The observation that discrete cell populations could be identified and isolated based on canonical marker expression enabled more contemporary prospective lineage tracing approaches, namely those making use of genetically engineered mouse models (GEMMs). Recombinase systems like *Cre*-*loxP*, which allow for cell-type specificity and intersectional spatial and temporal transgene expression control (3–5), constitute an essential tool for revealing the lineage history of newly identified cell types and refining the identities of some of the populations described above. Undoubtedly, one of the most valuable models for the identification of *Lgr5* as a bona-fide ISC marker was the *Lgr5*-*IRES*-*GFP*-*Cre^ERT2^* mouse (7). Similar strategies enabled the characterization of intestinal progenitor populations as well as the description of cell types that can aid intestinal epithelial regeneration under stress. For instance, it was demonstrated that *Dll1*^+^ cells in the crypt were secretory progenitors (66), and a combined lineage tracing/long-term label retention approach identified a subset of *Lgr5*^+^ ISCs as nondividing, secretory-biased cells (34). These label-retaining cells (LRCs), recently shown to depend on activation of the noncanonical Wnt pathway (36), can persist for days before differentiation into Paneth and EE subtypes, whereas goblet (and presumably tuft cells) are likely descendants of *Dll1*^+^ progenitors. The fact that some ISCs subsets can seemingly bypass the putative TA state to undergo direct secretory cell fate specification raises questions as to whether what we previously referred to as multipotent TA cells might in reality constitute an absorptive-lineage primed progenitor state, as these are remarkably proliferative. Interestingly, both *Dll1*^+^ and LRCs were recruited to aid in epithelial regeneration on loss of *Lgr5*^+^ ISCs (34, 66), a regenerative ability that was also demonstrated for *Alpi*^+^ enterocyte progenitors (67), EE cells (31), and even differentiated Paneth cells (68). Thus, it appears that postmitotic cells from both secretory and absorptive lineages can participate in intestinal epithelial regeneration, presumably upon exposure to the niche signals that are normally restricted to ISCs in the crypt. This is likely enabled by the epigenetic similarities between various crypt populations and ISCs (63, 69), as well as the presence of redundant niche sources (20, 21, 28, 70), which may allow more differentiated types to revert to the stem cell state with relative ease after encountering the right stimuli. Interestingly, under this model, one could argue that any devoted reserve stem populations would become dispensable; indeed, the most recent studies suggest that dedifferentiation (followed by subsequent upregulation of the ISC master regulator *Ascl2*) can satisfactorily explain intestinal epithelial regeneration upon initial loss of *Lgr5*^+^ ISCs (71, 72). However, this also constitutes a highly controversial issue, with radioresistant and other potential reserve stem cell populations remaining very possible, as these are not necessarily mutually exclusive models (73, 74). Altogether, these reports highlight the complexity of tissue regeneration and the need of much broader approaches to capture the injury-repair response (which involves the coordinated action of multiple cell types and biochemical axes) in its full magnitude.

### Single-Cell Transcriptomic Methods for Lineage Reconstruction in the Intestinal Epithelium

The above approaches have significant limitations. The reliance on prior knowledge invariably limits the scope of *Cre*-*loxP*-based GEMMs since known markers are required to develop suitable Cre drivers to perform such genetic fate mapping. Considering increasing evidence for cell-to-cell variation, any survey that relies on known markers will be limited in its distinction of cell subtypes and may fail to capture rare populations or intermediate states (53, 75). In addition, since these rely on the transfer of a label across generations, they invariably require cell division from the time of modification to the time of readout. Thus, subtle cell state transitions and quiescent cells in adult tissues will not be amenable to these approaches outside of, perhaps, a defined developmental window. Moreover, the majority of these methods require introduction of genetic modifications. To complicate matters, recent reports have shown that some of these GEMMs do not faithfully report transcriptional activity, as their reporter signals do not directly correlate with single-molecule mRNA in situ hybridization (64). Finally, in many instances, genetic modifications are technically challenging or not plausible to introduce, such as in human tissues.

A newer approach that overcomes some of these limitations employs scRNA-seq data to reconstruct lineages in an unbiased, marker-agnostic manner. This approach enables the study of the transcriptomes of individual cells at single-cell resolution to capture cellular heterogeneity compared with the bulk ensemble approach (76, 77). scRNA-seq has become instrumental for cataloguing cellular constituents across tissues in health and disease and has provided mechanistic insights into cellular function, dynamic processes like state transitions, and complex population-level responses (74, 78–82). Transcriptional profiles obtained through scRNA-seq also enable more accurate and robust identification of cell types and marker genes compared with the assessment of morphological characteristics or limited canonical marker expression (83–85). An ultimate extension of this is embodied by the concept of signaling entropy (86–88), which determines the degree of uncertainty, or differentiation potential, in a cell’s transcriptome by quantifying the relative activation levels of its molecular pathways as defined over an a priori specified protein interaction network. A more entropic gene expression profile (in which multiple lineage-specifying transcription factors are basally yet simultaneously active) is indicative of a higher developmental potential and phenotypic plasticity and may be used to assign stem cell identities a priori (87, 88).

Similarly, scRNA-seq can aid in the assembly of developmental trajectories (89), which are characterized by a progressive restriction in developmental potential and the concomitant acquisition of epigenetic, transcriptomic, functional, and morphological features characteristic of increasingly differentiated cell types. Assuming most developmental decisions are made gradually and accompanied by continuous changes in gene expression, cells can be computationally ordered into a developmental trajectory based on transcriptome similarity via trajectory inference. Although scRNA-seq only provides a static snapshot of a cell’s transcriptional state, analyzing thousands of cells undergoing these transitions at different stages of the developmental process should enable the capture of an entire lineage, now feasible by current scRNA-seq platforms that allow for the massively parallel sequencing of large numbers of individual cells. Transcriptionally similar cells can be plotted in two-dimensional space, in so-called pseudotime, to visualize how hierarchies are built during development or maintained during adulthood and help identify the gene expression changes, which occur during these transitions (90). However, resolving the correct lineage tree topology remains challenging, especially given that the available algorithms do not perform uniformly across datasets. Finally, although the majority of these algorithms rely on gene expression for their pseudotemporal analysis, it is worth noting that other strategies assess alternative transcriptome-readable biochemical parameters, such as the rate of mRNA splicing, to infer a lineage and importantly assign its directionality (91). Nonetheless, assumptions are made about steady-state kinetics and uniform rates of splicing across different genes.

The intestinal epithelium is particularly well-suited for scRNA-seq studies because it is a dynamic, self-renewing tissue whose vast cellular diversity and lineage relationships remain poorly understood (Figs. 1 and 3) (92, 93). Grün et al. devised an algorithm to identify rare cell types on scRNA-seq data acquired from intestinal organoids cultured ex vivo (33). Their group identified the major intestinal epithelial subtypes, including transitions through the TA state (characterized by high ribosomal gene expression) and three putative EE progenitor clusters. These scRNA-seq data were used to delineate continuous maturation trajectories, like those from enterocyte precursors to fully mature enterocytes, and identified *Reg4* as a new EE marker (33). *Reg4*^+^ EE cells were further distinguished based on Chromogranin A (*ChgA*) expression, with *ChgA*^high^ cells corresponding to serotonin (*Tph1*^+^)- and substance P (*Tac1*^+^)-producing enterochromaffin (EC) cells (33). Finally, they applied their algorithm to assess heterogeneity within *Lgr5*^+^ ISCs and their early progeny using an *Lgr5-eGFP-IRES-Cre^ERT2^;Rosa26-YFP* mouse model. They described different stages of Paneth cell maturation and reported the enrichment of *Lgr5* transcripts in early Paneth cells, pointing to this presumed R-spondin sensitivity as a potential mechanism through which a subset of this population reverts back to an ISC state upon injury (33, 69). Unlike other reports (34, 36), the authors assumed that the observed enrichment in Paneth/EE markers in part of the ISC clusters corresponded to rare populations of these secretory cells, concluding that *Lgr5*^+^ ISCs represent a homogeneous population (33). In a follow-up study (94), they presented a novel algorithm for guided lineage inference and applied it to a similar data set in which secretory cells emanated from a central, highly entropic ISC cluster as distinct branches, and enterocytes were seemingly connected to the ISCs via a TA population (94). Their interpretation underscores an emerging concept of TA cells as putative absorptive progenitors rather than true multipotent progenitors with multilineage potential (9, 35). By combining lineage tracing with a CD24-based enrichment strategy, the authors also demonstrated two distinct lineage trajectories for Paneth cells, one arising from *Dll1*^+^ common Paneth/goblet cell progenitors and another directly from ISCs/TA cells (94). This study supports an emerging model whereby direct specification of Paneth cells from ISC/TA can occur during homeostasis (34, 36). Taken together, these findings underscore the power of combining genetic lineage tracing strategies with single-cell transcriptomics to identify stem cells and progenitors and uncover alternative differentiation trajectories.

Recent reports have provided insight into cellular heterogeneity and spatial distribution within the gut epithelium. Haber and colleagues (83) profiled over 50,000 intestinal epithelial cells from wild type and *Lgr5-eGFP* knock-in mice with high granularity. Using unsupervised clustering methods, these studies organized major intestinal subtypes into multiple classes and stages of differentiation, derived their gene expression signatures, and identified novel candidate markers and regulatory transcription factors (83). From their sequencing results, Haber et al. also provided novel putative Paneth marker genes (like *Mptx2*) and identified two Paneth cell subtypes based on the differential expression of alpha-defensins, each differentially enriched along the proximal-distal axis (83). Of special interest was their characterization of enterocytes in up to seven stages of maturation. They described their differentiation trajectories in the proximal and distal small intestines and provided novel determinants of enterocyte fate (*Batf2*, *Mxd3*, etc.) and regional identity (*Jund*, *Osr2*, etc.) (83). This work was complemented by that of Moor and colleagues (95), who employed laser-capture microdissection (LCM) to characterize, at single-cell resolution, the transcriptomes of maturing enterocytes as they migrated along the crypt-villus axis. More than 80% (∼8,000) of the enterocyte-specific genes detected were zonated, and each maturation stage was associated with clearly demarcated functions (95). Enterocytes at the villus bottom were specialized in antimicrobial responses and expressed *Reg*-family genes and inflammasome components. As they migrated along the villus, their transcriptomes shifted to sequentially express carbohydrate, peptide, and fat absorption machineries, followed by purine-catabolic, immunomodulatory enterocytes at the villus tips (95). Unlike the historical view that considered the mature lineages of the intestine as static and postmitotic, these results added credibility to prior examples (96) of the plasticity of the transcriptomes of otherwise terminally differentiated cells, suggesting that cellular differentiation in the intestinal epithelium is a very dynamic process that starts in the crypts and spans to all the way along to the villus tips (22, 95, 97). These studies also exemplify how obtaining paired positional and transcriptomic information (spatial transcriptomics) (98) is critical to fully understanding lineage specification, as this is inextricably linked to the anatomical distribution of all these cell types along the crypt-villus axis.

EE cells comprise a rare but highly diverse secretory lineage devoted to sensing nutrient- and microbiome-derived metabolites, functioning as specialized signal transduction and hormone secretion units. Their hormone secretion profile has resulted in a “one-cell one-hormone” nomenclature by which they have been traditionally classified (99, 100). Haber et al. used scRNA-seq to corroborate that EE secretion profiles are more overlapping than traditionally acknowledged, supporting the need for a new nomenclature system where the concomitant expression of multiple hormones is considered (83). They also identified two different EC subtypes expressing distinct *Reg4* levels (83), lending additional support to *Reg4* as an EE marker (33). The investigators additionally described two tuft progenitor populations and two mature subtypes, one enriched in neural development genes (tuft-1) and the other in genes specific for the immune response (tuft-2) (83). However, they did not address the developmental origin of these cells. To address controversies regarding tuft cell ontogeny and *Atoh1* dependence, Herring et al. developed the p-Creode algorithm, which incorporates data modalities as variate as mass spectrometry, cell imaging, and scRNA-seq for lineage inference (101). They conclude that tuft cells are specified separately from other secretory subtypes, sharing a common trajectory with enterocytes in the small intestine and arising independently near the ISCs in the colon (101). Additional LOF experiments demonstrated *Atoh1* dispensability and the potential nonsecretory origin of tuft cells in the small intestine but highlighted the necessity of *Atoh1* in the colon (101). This study also investigated the developmental origin of colonic *Reg4*^+^ deep crypt secretory cells, localizing them along the goblet cell trajectory (101).

The study of injury-induced regeneration has also benefited from single-cell transcriptomics, especially for reconciling the multiple reported ISC populations. Comparative bulk RNA-seq analysis of putative ISC populations suggested that *mTert*^+^ and *Bmi1*^+^ cells isolated from reporter mice shared EE characteristics and were indeed transcriptionally distinct from *Lgr5*^+^ ISCs (31). Further analysis by scRNA-seq supported the heterogeneity of *Bmi1*^+^ cells not as putative ISCs but rather as postmitotic EE subsets distinguished by their hormone-expression profiles (31). These findings were underscored by Shivdasani and colleagues, who similarly concluded that *Bmi1*^+^ cells were EE precursors that can adopt an ISC-like epigenetic state on injury (69). *Bmi1*^+^ cells shared similarities to LRCs (34) and *Dll1*^+^ progenitors (66) but lacked *Neurog3* transcripts and Paneth/goblet markers, consistent with EE lineage-restricted cells that surpassed the earliest stage of EE specification. Since they coexpress hormones associated with discrete EE subsets, *Bmi1*^+^ cells represent a committed, multicapable EE progenitor at incipient stages of differentiation (31). Using *Prox1* as an orthogonal marker of EE cells, scRNA-seq identified subsets with partial CBC transcript enrichment and mixed EE/tuft cell signatures, pointing to the existence of a shared common progenitor between the EE and tuft cell lineages (31). Additionally, *Prox1*^+^ cells were capable of long-term multilineage differentiation (31), a phenomenon similarly observed in *Bmi1*^+^ cells (59) and also accentuated after irradiation (31, 59). These studies support a plasticity model in which postmitotic, committed/differentiated populations enriched in EE markers and devoid of the *Lgr5*^+^ ISC signature possess injury-inducible stem cell activity (31, 63).

In addition to reconciling putative ISC identities, scRNA-seq has also been used to discover and characterize additional subpopulations that become activated on damage, as well as the transcriptional programs that underlie the mechanisms of injury-induced regeneration. One of these corresponds to a population expressing high levels of *clusterin* (*Clu*), referred to by Ayyaz et al. as revival stem cells (revSCs) (74). These were identified by scRNA-seq analysis of the regenerating intestinal epithelium 3 days postirradiation as a rare, *Lgr5*^−^, YAP-dependent injury-induced quiescent cell type (74). Transgenic *Clu* reporter mice confirmed limited numbers of *Clu*^+^ cells during homeostasis, rarely localized in the crypt. Following irradiation, revSCs crypt localization was widespread, with *Clu*^+^-derived epithelium massively repopulating the small intestine and colon and giving rise to all the lineages over time (74). Crypts containing revSCs lacked *Lgr5* and *Olfm4* expression, indicating these do not overlap with bona-fide ISCs (74). Consistent with this finding, ablation of *Clu*^+^ cells cooccurred with no detrimental phenotype under homeostasis; however, impaired epithelial regeneration was observed following irradiation and colitis (74). Interestingly, even though these cells are derived from *Lgr5*^+^ ISC progeny, their exact position within the lineage is poorly known.

A similar study characterized an alternative injury-inducible population: a class of cells that revert to a fetal-like, *Sca1*^+^, Wnt-independent program to initiate epithelial regeneration after damage (102, 103). Shortly after parasitic helminth infection and radiation injury, *Sca1*^+^ cells form regenerative, primarily undifferentiated, proliferative crypts devoid of canonical ISC markers. By the time *Lgr5*^+^ ISCs reemerge and repopulate the regenerating epithelium, *Sca1* expression decreases (102). Importantly, *Sca1*^+^ cells are part of the *Lgr5*^+^ ISC lineage in the adult small intestine, but they can arise and regenerate the epithelium independently of *Lgr5*^+^ ISCs themselves and the Wnt/Rspo niche that is required for adult tissue maintenance (102). The evidence of differentiated cells coopting a fetal developmental program to repair adult tissue is remarkable and already reported in similar contexts, which points to an idea of rederiving stem cell identities in the same way the tissue was originally formed (103–105). Still, the degree of overlap between *Sca1*^+^/*Clu*^+^ and the other reported regenerative populations remains unclear (73, 92).

### Combination Approaches to Study Lineage in the Intestinal Epithelium

There are limitations to the use of single-cell technologies that preclude our studies of cell identities and lineage relationships. Besides the major issues of gene and population dropout, some other problems associated with scRNA-seq involve the introduction of potentially ambiguous cell identities and the inherent difficulty in detecting delicate transitions between discrete fates. Furthermore, these are largely destructive methods that lack spatial resolution. Spatial understanding of self-renewal and differentiation are essential because of the anatomic localization of the ISC niche and the signaling gradients that promote differentiation (Fig. 1) (17, 19–22, 98). The lack of consistency across published lineage reconstruction algorithms is also potentially problematic; many of these require extensive (nonjustified or arbitrary) parameter tweaking and only work well for the data set on which they were developed. Finally, none of these approaches proves true genetic relationships between cells. For this reason, unless combined with some type of genetic mapping strategy, they all require extensive experimental validation. As a further complication, the passage of cells along developmental trajectories is rarely synchronous, and population-level analyses rarely provide sufficient resolution to dissect true cellular diversity or properly allocate lineages. Therefore, methods that pre-enrich for a population of interest or adopt experimental designs that enhance temporal resolution are likely biased but should still be useful to complement scRNA-seq studies.

One recent study by Gehart et al. demonstrates the effectiveness of combining temporally-resolved genetic labeling with scRNA-seq-based lineage reconstruction (106). *Neurog3* is a transcription factor essential for the EE program and its expression pattern is spike-like in EE progenitors with rapid drop as further differentiation ensues. The authors coupled *Neurog3* expression to a dual-fluorescence time reporter consisting in a fast-folding, destabilized mNeonGreen protein followed by a second, stable, and slow-folding tdTomato fluorophore translated at an equimolar ratio. Using this reporter, the authors were able to sort populations based on distinct fluorescence emission and intensity profiles, obtaining a real-time, ordered differentiation trajectory of the EE lineage where the ratio of green and red is an estimate of the time elapsed since EE fate specification. By overlaying scRNA-seq profiles on this molecular clock, they generated an experimentally derived EE developmental trajectory and associated gene expression signatures (106). Their analysis also revealed remarkable plasticity across mature EE subpopulations and refined aspects of EC differentiation (106). Whereas the differential hormone expression found in EC cells at the crypt (*Tac1*^high^, *Sct*^−^) versus the villus (*Tac1*^low^, *Sct*^+^) was previously ascribed to the existence of parallel lineages, their results suggested these two populations arise sequentially rather than in parallel, supporting a model where *Tac1*^high^ cells constitute a link between progenitors and *Sct*^+^ EC cells (106). These results added further depth to the description of bone morphogenetic protein (BMP) gradients shaping the EE hormonal expression profile along the crypt-villus axis (97), suggesting that the different EE lineages reported to date are in actually different niche-induced states within the same lineage (9). Similarly, their data refuted previous observations that considered *Reg4*^+^ and *Reg4*^−^ EC cells as separate lineages (83). Their study also raised questions about how differentiated *Bmi1^+^* and *Prox1^+^* cells are along the EE lineage (31), as their data suggest *Bmi1* and *Prox1* are expressed earlier than in a terminally differentiated state. In summary, Gehart and colleagues provide a map of the intestinal EE cell differentiation landscape that will inform the field in identifying relevant transiently expressed regulators. This study also highlights the difficulty of inferring lineage trajectories and lineage relationships based on transcriptomic profiles alone and underscores the synergism of combining experimental labeling and computational approaches.

## CONCLUDING REMARKS

Most of our current knowledge on the relationships between the ISCs and their progeny is still grounded on early studies using genetic manipulations and cell signaling perturbations. However, these methods have failed to provide a consensus view on the molecular identities of all these cell types and how fate decisions are made along intermediate states. The model generated from these approaches is that of an intestinal epithelial lineage tree with a single trunk leading in a unidirectional manner to only a few, poorly defined branches. Prospective lineage tracing strategies using defined marker genes have helped reveal the intestinal epithelial lineage tree one-cell population at a time, yet they are also not free of limitations as the specificity of these marker genes is often confounding. Although the identity and behavior of these intermediate states still remain to be fully elucidated, novel scRNA-seq approaches have enabled the interrogation of large developmental continuums at unprecedented levels of detail and in a largely unbiased manner, refining our knowledge of the intestinal epithelial lineages (Fig. 3) (31, 33, 36, 74, 83, 94, 95, 101, 102). With new cell atlas studies, gene expression signatures for many of the mature and developing intestinal epithelial populations have been published, new intermediary secretory and functional absorptive subtypes have been defined, the classification of EE and tuft cells has diversified, and important regional differences across subtypes have been reported. Furthermore, these findings are aiding studies of intestinal epithelial regeneration. A key message from these studies is that the cellular diversity of the intestinal epithelium is more vast than previously envisioned. In addition, there is a growing realization that single canonical marker expression fails to characterize the diversity of otherwise well-studied stem and progenitor populations, underscoring the need of multidimensional data for appropriate cell classification. Interestingly, scRNA-seq is challenging existing paradigms and revealing that features like multilineage differentiation may need to be explained at the population level, as individual stem cells or progenitors within a pool may display a predetermined bias for differentiating toward one lineage over others (36, 107, 108). Thus, it appears clear that *Lgr5*^+^ ISCs are neither homogeneous nor equipotent, and that their fates are intertwined with extrinsic signals from the niche. This heterogeneity, perhaps overlooked due to reliance on *Cre-loxP* approaches where clonal resolution becomes lost (7, 53, 58), underscores the importance of actual clonal analysis and the need to incorporate spatial information in our lineage tracing endeavors.

The high granularity of single-cell approaches poses its own challenges and raises new questions as to what constitutes a transient cell state versus a discrete cell population, as well as the significance of the distinctly clustered single-cell populations seen in many of these transcriptomic studies. The latter relates to clustering being as much of an art as it is a science. What defines a functional state versus a new cell type? How many of these finer granularity cell-type descriptions are biologically relevant? How do we approach the inconsistencies found in clustering algorithms, and how do we decide which one is more suited to our data set? Thus, scRNA-seq data lacks meaning in the absence of experimental validation. Interestingly, genetic labeling and computational lineage inference can be combined, allowing us to address complex questions that could otherwise not be tackled separately (106, 109–112). Therefore, rough lineage trajectories may be described based on the initial transmission of a genetically heritable mark and then refined and annotated using single-cell transcriptomics. Recent approaches leverage a combinatorial increase in label diversity to gain single-cell resolution using heritable genetic barcodes, some of which are mutable or evolve within genetically encoded CRISPR/Cas9 arrays that can even spread across the multiple lineages of entire organisms (53, 110). When these genetically inheritable barcodes are transcribed, single-cell transcriptomics can be used for concomitant cell type identification and barcode retrieval to identify genetically supported, clonal lineage relationships (113–117). Although potentially promising, none of these approaches have been applied to interrogate the lineage relationships of the adult intestinal epithelium yet. Still, we anticipate this will open the door to new and exciting discoveries in the field. One can also anticipate that other “-omic” measurements will come into play, especially those using epigenetic information (118), as these may anticipate changes in gene expression.

Finally, lineage tracing in the context of experimental perturbations (e.g., cell-type specific ablation, genetic perturbations, or pharmacological modulation of cell-cell signaling) may reveal how lineages are structured as a function of gene regulation and signal transduction (16, 56). This is grounded in our belief that homeostasis is sometimes best understood following perturbation of the steady state (16). Our ability to grow intestinal epithelial organoids in culture is also helping advance lineage reconstruction endeavors, as these constitute a readily accessible platform that facilitates batched and controlled biochemical perturbation of multiple signaling pathways simultaneously (119, 120). Organoids have also been used in combination with lineage tracing and scRNA-seq-based lineage inference to aid our understanding of new putative stem/progenitor cell populations, the injury-repair process, and disease (73, 121, 122). Studies of disease states are facilitated by the generosity of patients undergoing surgery and/or endoscopic procedures and by large biobanking efforts, which provide us with valuable human primary tissues to help us better understand human intestinal biology. Thus, single-cell technologies are tools in our arsenal that can be used combinatorially with other approaches. The possibilities are endless, and we need to use them to address the right questions.

## GRANTS

C.C. is supported by a predoctoral NYSTEM training grant and J.M. is supported by T32 training grant 5T32DK083256-12. P.A.S. is supported by R01NS103473. K.S.Y. is supported by Burroughs Wellcome Fund Career Award for Medical Scientists, Irma T. Hirschl Trust, Louis V. Gerstner Jr. Scholars Award, Lisa Dean Moseley Foundation, NIH 1DP2DK128801, and 1R01AG067014 awards.

## DISCLOSURES

No conflicts of interest, financial or otherwise, are declared by the authors.

## AUTHOR CONTRIBUTIONS

C.C. and K.S.Y. prepared figures; C.C., P.A.S., and K.S.Y. drafted manuscript; C.C., M.T., J.M., T.A., P.A.S., and K.S.Y. edited and revised manuscript; P.A.S. and K.S.Y. approved final version of manuscript.
